# Parents’ Perception and Use of Skin-to-Skin Care in Jeddah, Saudi Arabia: A Cross-Sectional Study

**DOI:** 10.7759/cureus.56734

**Published:** 2024-03-22

**Authors:** Ahmad Ismail, Amnah Mahdi, Karimeh M Al-Nuaimi

**Affiliations:** 1 Nursing, Fakeeh College for Medical Sciences, Jeddah, SAU

**Keywords:** saudi arabia, parents, fathers, perception, kangaroo care, skin-to-skin care

## Abstract

Background: Skin-to-skin care (SSC) between newborns and their parents provides many positive outcomes for both newborns and their parents. However, there is a lack of research assessing the perception of parents, especially fathers, of SSC in Saudi Arabia.

Purpose: The aim of the study is to assess parents’ perception of SSC in Saudi Arabia.

Methods: This cross-sectional study used an online survey. Data were collected from a convenience sample of 140 parents of hospitalized neonates between January and June 2023. Data were collected from one private hospital (Dr. Soliman Fakeeh Hospital) and one public hospital (King Abdulla Medical Complex) in Jeddah, Saudi Arabia. The parents' perceptions of SSC were assessed using the Parents' Perceptions of SSC tool. An independent t-test was used to compare mothers and fathers in their perceptions of SSC.

Results: One hundred and forty parents completed the online survey (70 females and 70 males). The majority of the parents did not use SSC with their babies (n=102, 73%), did not read or hear about the use and benefits of skin-to-skin care (n=100, 71%), and did not receive information or training on SSC from healthcare professionals (n=112, 80%). Mothers’ perception of SSC was significantly higher than that of fathers (*p *≤ 0.05).

Conclusion: Fathers’ perception of SSC was lower than that of mothers. Awareness and training programs are needed to inform parents, especially fathers, regarding SSC and its benefits in Saudi Arabia.

## Introduction

Neonatal morbidity and mortality are major health problems, especially in low- and middle-income countries. Worldwide, 2.4 million infants died in the neonatal period (first month of life) in 2020, accounting for 47% of all deaths of children less than five years old [1]. Neonates are at risk for many health-negative consequences after birth, such as hypothermia [2], hypoglycemia [3], infection [4], pain [5], sleep problems (especially in the Neonatal Intensive Care Units (NICUs)) [6], and stress (especially hospitalized neonates who are separated from mothers) [7].

Skin-to-skin care (SSC) between newborns and parents provides many physical and psychological positive outcomes for newborns and their parents [8]. SSC is the initiation/maintenance of contact between the infant and the mother's or father's chest. It is usually performed by mothers with exclusive breastfeeding [9-12]. Several studies have shown the positive outcomes of SSC. SSC is a cost-effective intervention that may reduce neonatal mortality and morbidity. It reduces the rate of nosocomial infection, improves the neonatal physiological stability and cardiorespiratory functions, reduces the risk of hypoglycemia and hypothermia, reduces the neonatal pain level, promotes the neonatal growth, enhances the infant cognitive development and executive functions, improves the neonatal sleep, and increases the rate of exclusive breastfeeding [7,8,13-18]. SSC promotes the quality of mother-infant relationships, decreases maternal postpartum depressive symptoms, reduces maternal stress and anxiety, enhances maternal bonding and attachment behavior, and improves the quality of maternal caregiving [14,19,20]. Early SSC reduces the incidence of postpartum hemorrhage by shortening the third-stage labor duration [21-24]. Many institutions and organizations recommend SSC, especially after birth [9,25], which can be applied in all contexts (from low-resource to high-technological contexts) [25]. In most cases, the primary beneficiaries of perinatal care (including SSC) are the mothers and their newborns.

Most research on SSC has concentrated on mothers more than fathers [26]. However, the notion that mothers spend more time caring for their infants than fathers is changing, especially in Western societies [27,28]. Fathers spend much more time caring for their children than in the past [29]. Several studies showed that fathers’ behaviors and caring for their infants result in positive developmental outcomes such as attachment, speech, and play [28,30]. Fathers can also practice SSC with their neonates [28]. Research revealed that SSC with fathers leads to positive outcomes for infants and fathers [31,32]. Many factors influence the father’s involvement in neonatal care such as gender role and socio-cultural beliefs [33]. Some fathers believe that infant care is traditionally a female role [33]. The willingness of fathers to practice SSC care with their neonates is affected by their perception and beliefs about SSC. In Saudi Arabia, the SSC rate is low [8]. A recent study found that parents in Saudi Arabia exhibit positive behavior toward SSC. However, this study included only one father (2%) out of 51 participants [34]. There is a lack of research assessing the fathers’ perception and practice of SSC in Saudi Arabia. Therefore, this study aimed to assess parents’ perception of SSC, especially fathers in Saudi Arabia.

## Materials and methods

Study design

This research utilized a cross-sectional online survey to collect data from parents regarding their perception of SSC. An equal number of mothers and fathers were recruited. Data were collected between January and June 2023. 

Study setting

The current online survey was shared with parents in one private (Dr. Soliman Fakeeh Hospital) and one public (King Abdulla Medical Complex) hospital in Jeddah, Saudi Arabia, who had neonates hospitalized in the hospital units or the outpatient neonatal clinics seeking care and follow-up.

Sample and sampling

The sample of this study was the parents of neonates who received care at hospital units or outpatient clinics. As this study aimed to assess and compare the perception of fathers and mothers regarding SSC, the number of required parents was calculated based on the independent t-test using power analysis software (G*Power) [35]. With a moderate effect size, an alpha level of 0.05, and a power of 80%, the minimum required number of parents was 128 (64 fathers and 64 mothers). However, we were able to recruit 140 parents (70 fathers and 70 mothers). 

Data collection tools

The instrument of this study was developed and administered in Arabic (the mother language of parents). It consisted of three parts. The first one included the demographic characteristics of the parents, such as age and gender. The second part included items related to SSC and awareness, such as whether the parents had previously used SSC for their babies and whether they had heard or read about SSC. The third section included 19 items that assessed the parents' perception of SSC. These items were measured using a 5-point Likert agreement scale (1= Strongly Disagree to 5= Strongly Agree). The authors developed the instrument for the purpose of this study. It was developed based on the following literature: studies by Al-Matary et al. (2022) [34], Chan et al. (2016) [10], Olsson et al. (2017) [25], and Seidman et al. (2015) [36]. The instrument was then validated by a panel of six experts in nursing, research, neonatal care, and maternity care. The content validity index was high (0.92). The internal consistency reliability using Cronbach’s Alpha was high (0.98).

Data collection procedure

After obtaining ethical approval from Fakeeh College for Medical Sciences (378/IRB/2022) and the Ministry of Health of Saudi Arabia (A01580), the principal investigators arranged an initial visit to the target settings. They met with the nursing managers of the neonatal units. The primary purpose of the study, expected outcomes, and clinical implications were explained. The managers were informed to instruct parents to fill out one survey per family to avoid any dependence on the answer in case of having a father and mother for the same baby. The survey link and QR code were shared with the nursing managers who distributed them to the parents. The first page of the survey included information on the study and the consent to participate option. If a participant decided to participate, she/he clicked on the “agree to participate” button, where the platform moved her/him to the survey items. If the participant did not agree to participate, she/he clicked on the “disagree to participate” button, and then the platform thanked and exited her/him. Completion and submission of the survey implied consent to participate. The principal investigator visited the hospitals biweekly for follow-up and to answer any questions from the nursing managers.

Data analysis

IBM SPSS Statistics for Windows, Version 26 (Released 2019; IBM Corp., Armonk, New York, United States) was used to analyze the data. The collected data were exported from the SurveyMonkey platform to the SPSS. Data were coded, entered, and analyzed. Descriptive statistics using mean and standard deviation were used to summarize the continuous variables. Frequencies and percentages were used to summarize the categorical variables. The perception of parents of SSC was computed to generate one mean and one standard deviation reflecting the whole status of the parents’ perception towards SSC. The independent t-test was used to compare the perceptions of fathers and mothers regarding SSC. Chi-square and Fisher exact tests were used to compare fathers and mothers in their demographic characteristics. Results were considered significant when the p-value was less than 0.05.

Ethical considerations

Completion and submission of the survey implied consent to participate. The study was voluntary and anonymous. Data were collected using the SurveyMonkey Platform, which has security measures in place to protect the confidentiality of the participant’s data. Participants’ data will be destroyed by secured deletion five years after the publication of the results. Data were stored using a password-protected file. Data were stored at the principal investigator’s institution.

## Results

One hundred forty parents completed this online survey. Half of the parents were males (50%), and half were females (50%). The mean age of the parents was 35 ± 7. The majority of the parents were from private hospitals (86%), Saudi (84%), employed (76%), and held a bachelor’s degree (69%). Near half of the participants had a monthly income of less than 10,000 SR (59%). The majority of the infants were more than 37 weeks of age (93%). Near half of the infants were admitted into the wards (53%). There was a significant difference between fathers and mothers in terms of educational level, employment, and income. Fathers had more master’s degrees, more employment, and higher income (p ≤ 0.05) (Table 1).

**Table 1:** Parents' demographic characteristics (n= 140) *: Significant; F: Frequency; %: Percentage; NICU: Neonatal Intensive Care Unit

Character	Male and Female Parents		Male Parents		Female Parents		p-value
	F	%	F	%	F	%	
Citizenship							
Saudi	118	84%	56	40%	62	44%	0.16
Non-Saudi	22	16%	14	10%	8	6%	
Education							
High school or less	16	11%	8	6%	8	6%	0.001*
College diploma	4	3%	4	3%	0	0%	
Bachelor	96	69%	38	27%	58	41%	
Master and above	24	17%	20	14%	4	3%	
Employment							
Employed	106	76%	0	0%	34	24%	0.001*
Unemployed	34	24%	70%	100%	36	26%	
Monthly income							
0-10000 SR	82	59%	24	17%	58	41%	0.001*
10001-20000 SR	28	20%	24	17%	4	3%	
More than 20000 SR	30	21%	22	16%	8	6%	
Hospital							
Private	120	86%	58	41%	62	44%	0.334
Public	20	14%	12	9%	8	6%	
Unit where the infant was							
Outpatient	48	34%	30	21%	18	13%	0.051
Ward	74	53%	34	24%	40	29%	
NICU	14	10%	6	4%	8	6%	
Emergency	4	3%	0	0%	4	3%	
Infant age at birth							
Less than 37 weeks	10	7%	6	4%	4	3%	0.51
37 Weeks or more	130	93%	64	46%	66	47%	

The majority of parents did not use SSC with their babies (73%), did not read or hear about the use and benefits of SSC (71%), and did not receive information or training on SSC from healthcare professionals (80%). More male parents received information or training about the SSC from healthcare professionals than female parents (20 vs 8, p ≤ 0.05) (Table 2).

**Table 2:** Parents’ awareness and use of SSC (n= 140) *: Significant; F: Frequency; %: Percentage; SSC: Skin-to-skin care Statistical test used: Chi-square test

	Male and Female Parents		Male Parents		Female Parents		p-value
Item	F	%	F	%	F	%	
Heard or read about the use and benefits of SSC							
Yes	40	29%	20	14%	20	14%	1.0
No	100	71%	50	36%	50	36%	
Received information or training on SSC from healthcare professionals							
Yes	28	20%	20	14%	8	6%	0.01*
No	112	80%	50	36%	62	44%	
Used the SSC with the baby							
Yes	38	27%	20	14%	24	17%	0.29
No	102	73%	50	36%	46	33%	

Mothers had more positive perceptions toward SSC than fathers (mean=4.1 vs 3.6; p ≤ 0.05). Mothers’ perception was higher than fathers’ in all items, which was significant in 9 out of 19 items (p≤ 0.05). There were significant differences in the following items: 1) SSC improves the physical health of newborns, 2) Kangaroo increases the verbal and emotional bonding between the father/mother and the newborn, 3) SSC creates a sense of security in the newborn, 4) SSC enhances the mother's or father's love of the newborn, 5) SSC reduces the stress of the father or mother, 6) SSC provides warmth for the newborn, 7) SSC is important to improve health care outcomes of newborns, premature infants, and infants, 8) Father can practice SSC for the newborn, and 9) In order to practice kangaroo baby care, I need to maintain my privacy (p ≤ 0.05) (Table 3).

**Table 3:** Parents' perception of SSC (n = 140) *: Significant; t: independent t-test; SSC: skin-to-skin care Likert agreement scale rating (1: Strongly Disagree, 2: Disagree, 3: Neutral, 4: Agree, and 5: Strongly Agree)

No	Item	Fathers’ Mean out of 5 (n = 70)	Mothers’ Mean out of 5 (n = 70)	t	p
1	SSC improves the physical health of newborns.	3.5	4.1	2.5	0.01*
2	SSC makes the father or mother take better care of the baby.	3.6	3.9	1.1	0.26
3	SSC increases the verbal and emotional bonding between the father/mother and the newborn.	3.4	4.3	2.6	0.01*
4	SSC creates a sense of security in the newborn.	3.9	4.3	2.1	0.04*
5	SSC enhances the mother's or father's love of the newborn.	3.7	4.4	2.5	0.01*
6	SSC reduces the stress of the father or mother.	3.7	4.3	2.4	0.02*
7	SSC provides warmth for the newborn.	3.7	4.4	3.1	0.01*
8	SSC is important to improve health care outcomes of newborns, premature infants and infants.	3.7	4.3	2.6	0.01*
9	SSC reduces the newborn length of stay at the hospital after birth.	3.6	3.9	1.4	0.15
10	SSC improves newborn circadian rhythm.	3.6	3.7	0.5	0.1
11	SSC improves newborn immunity.	3.5	3.8	1.3	0.20
12	SSC improves the growth of newborns.	3.6	3.9	0.95	0.34
13	SSC improves the blood oxygen level in the newborn.	3.7	3.8	0.5	0.6
14	SSC regulates the newborn heartbeat.	3.7	4.0	1.3	0.19
15	SSC improves newborn breathing.	3.7	3.9	1.2	0.24
16	Father can practice SSC for the newborn.	3.5	4.1	2.9	0.004*
17	Newborn medical conditions may hinder the SSC*.	3.2	3.4	0.60	0.50
18	Encouragement of medical and nursing staff to practice SSC has a significant impact on the practice of SSC.	3.6	4	1.7	0.09
19	In order to practice SSC, I need to maintain my privacy.	3.5	3.9	2.1	0.04*
	Total	4.1	3.6	2.03	0.04*

## Discussion

The majority of parents did not use SSC with their babies, did not read or hear about its use and benefits, and did not receive information or training on SSC from healthcare professionals. Mothers showed more positive perceptions of SSC than fathers. This study highlights two main points for discussion. The first one is that SSC is not used adequately by parents regardless of gender. The second point is that mothers have higher positive perceptions of SSC care than fathers in Saudi Arabia.

Worldwide, several studies have assessed the positive impact of SSC on the outcomes of babies and fathers [31,37-39]. A study examining the effect of SSC between fathers and newborns on the attachment relationship in northern Taiwan found a higher significant attachment score (measured by the Father-Child Attachment Scale) in fathers who practiced SSC compared to those who did not [37]. A review of 12 studies on the impact of father-infant SSC on infant and paternal outcomes found that father-infant SSC resulted in several positive infant and parental outcomes, such as temperature and pain control of infant, positive behavioral responses of infant, improved parental attainment and interaction, and reduced paternal stress and anxiety [31]. Another qualitative study from Australia showed that fathers perceived positive experiences of SSC in the NICU setting. Fathers perceived SSC enhanced their bonding and attachment with their babies [38]. Another study from Turkey found that fathers' SSC improved babies' physiological parameters and stability [39]. Previous studies demonstrate that fathers can perform SSC even in critical care settings, which results in positive outcomes. However, in this study, the rate of SSC by both fathers and mothers was low.

Our study shows that the majority of parents did not use SSC with their babies. This is consistent with recent research showing that SSC is not adequately used by parents in Saudi Arabia [8]. The perception of parents of SSC was assessed primarily from the perspective of mothers in Saudi Arabia [34,40]. A recent study was conducted in Saudi Arabia in 2022 to evaluate parents' perception of SSC care with their neonates. Small, unequal groups of 51 parents participated in this study (50 mothers and only one father). Most of the items of the study (9 out of 17) asked about the parents’ perception of healthcare providers' role in SSC. The study found that the perception of parents' score of SSC was 75%. The study also found a higher significant difference in the perception of parents with female children than those with male children [34]. The same study revealed that the majority of the parents heard about SSC (88%), perceived that SSC provides warmth to the neonates (82%) and reduces the duration of hospital stay (88%), and received information about SSC from healthcare professionals (90%). In our study, we recruited equal groups and a larger sample of 140 parents (70 mothers and 70 fathers). The items in our study focused on the parent’s perception of the outcomes of the SSC. Our study found a more positive perception of SSC (82%) than the previous study. The majority of parents in our study perceived that SSC provides warmth to the babies and reduces the duration of hospital stays. Our study found that most parents did not read or heard about SSC and did not receive information or training on SSC from healthcare professionals. The previous study did not ask the parents about their practice of SSC [34], while our study found that the majority of parents did not practice SSC with their neonates despite their positive perception. The results of our study are also consistent with the results of another recent study conducted in Saudi Arabia to assess the SSC rate and mothers’ perceptions of immediate SSC after vaginal birth in Jeddah, Saudi Arabia. The study found that only 15% of mothers practiced SSC. The perception of mothers of SSC was positive (67%). Some mothers also felt positive about their experience of SSC, and some felt overwhelmed [40]. In our study, more parents practiced SSC (27%) and reported more positive perceptions of SSC (82%) than in the previous study.

Mothers in this study reported more positive perceptions of SSC than fathers. This may be attributed to the father’s traditional role where the father is primarily responsible for work and earning income for the family. Mothers are responsible for household chores and childcare. A recent research study from Malawi revealed that the traditional father's role influences fathers’ involvement in caring for hospitalized newborns. The father is responsible for the family’s needs, and the mother is responsible for the infant's care [33]. More research is needed to identify the factors influencing parents’ practice of SSC in Saudi Arabia. Research is also needed to identify why fathers in Saudi Arabia have a less positive perception of SSC than mothers.

This study provided significant information on parents' perceptions of SSC in Saudi Arabia, especially from fathers' perspective. It compared fathers and mothers using equal groups of 70 fathers and 70 mothers of hospitalized infants. This study also used specific items to assess the perception of fathers and mothers of the outcomes of the SSC and their practice. These items were constructed based on previous tools and literature, and a panel of experts validated them. However, this study has many limitations. The study was conducted in two hospitals in Jeddah, Saudi Arabia, and may not be generalizable to other areas. Additionally, the study relied on self-reporting from the participating parents, which may introduce potential inaccuracies. Future research should consider recruiting parents from other cities in Saudi Arabia and using a combination of objective and subjective measures to better understand parents’ perceptions and experiences of SSC.

## Conclusions

Most parents did not use SSC with their babies, did not read or hear about the use and benefits of the SSC, or received information or training on SSC from healthcare professionals. Mothers showed more positive perceptions toward SSC than fathers. Healthcare professionals should educate and encourage parents to practice SSC with their babies. Awareness programs at the level of neonatal departments are needed to encourage and inform parents, especially fathers, regarding SSC and its benefits in Saudi Arabia. More research is needed to explore why fathers have lower perception scores than mothers regarding SSC.
